# Primary Cilia as Signaling Hubs in Health and Disease

**DOI:** 10.1002/advs.201801138

**Published:** 2018-11-16

**Authors:** Yuhei Nishimura, Kousuke Kasahara, Takashi Shiromizu, Masatoshi Watanabe, Masaki Inagaki

**Affiliations:** ^1^ Department of Integrative Pharmacology Mie University Graduate School of Medicine Tsu Mie 514‐8507 Japan; ^2^ Department of Physiology Mie University Graduate School of Medicine Tsu Mie 514‐8507 Japan; ^3^ Department of Oncologic Pathology Mie University Graduate School of Medicine Tsu Mie 514‐8507 Japan

**Keywords:** cancer, ciliopathy, obesity, receptor tyrosine kinases, ubiquitin‐mediated protein degradation

## Abstract

Primary cilia detect extracellular cues and transduce these signals into cells to regulate proliferation, migration, and differentiation. Here, the function of primary cilia as signaling hubs of growth factors and morphogens is in focus. First, the molecular mechanisms regulating the assembly and disassembly of primary cilia are described. Then, the role of primary cilia in mediating growth factor and morphogen signaling to maintain human health and the potential mechanisms by which defects in these pathways contribute to human diseases, such as ciliopathy, obesity, and cancer are described. Furthermore, a novel signaling pathway by which certain growth factors stimulate cell proliferation through suppression of ciliogenesis is also described, suggesting novel therapeutic targets in cancer.

## 1 Introduction

Primary cilia are nonmotile, 1–10 µm long antenna‐like structures observed in a variety of vertebrate cells. Primary cilia detect extracellular cues, such as mechanical flow and chemical stimulation, and transduce these signals into the cell.^1^, ^2^, ^3^, ^4^, ^5^, ^6^, ^7^ Therefore, the dysregulation of primary cilia can cause various diseases, including congenital anomalies, neurodevelopmental disorders, obesity, and cancer.^8^, ^9^, ^10^, ^11^, ^12^, ^13^, ^14^

**Figure 1:**
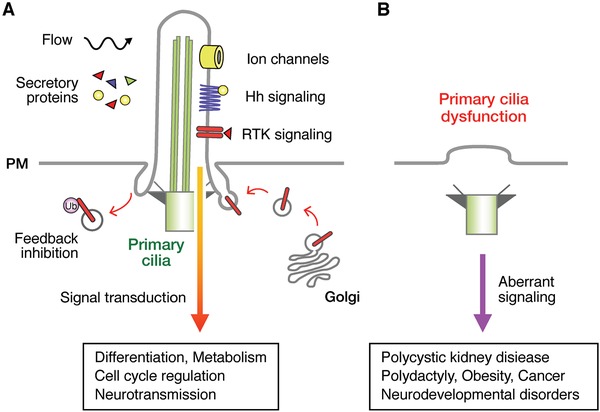
Simplified overview of growth factor and morphogen signaling through primary cilia. A) Primary cilia detect extracellular cues, such as secreted growth factors and morphogens, through their receptors and mediate their signaling to regulate a wide range of activity, including differentiation, cell cycle regulation, metabolism, and neurotransmission. B) Signal transduction processes are impaired when primary cilia are dysfunctional, causing various diseases, such as ciliopathy, obesity, cancer, and neurodevelopmental disorders.

The signaling of growth factors and morphogens is also mediated by primary cilia^15^, ^16^, ^17^, ^18^, ^19^, ^20^, ^21^, ^22^, ^23^, ^24^, ^25^, ^26^, ^27^, ^28^, ^29^ (**Figure** **1**). The receptors for these growth factors and morphogens are often localized in primary cilia. When these molecules bind to their receptors, various combinations of intracellular signaling pathways are activated.^30^ In receptor tyrosine kinase (RTK) signaling, the binding of a growth factor to its receptor usually leads to dimerization and/or oligomerization of the receptors, resulting in the *trans*‐autophosphorylation of multiple tyrosine residues. *Trans*‐autophosphorylation releases *cis*‐autoinhibition, which activates various downstream signaling proteins containing Src homology‐2 (SH2) or phosphotyrosine‐binding (PTB) domains. These proteins bind to the autophosphorylated RTKs through their SH2 or PTB domains and propagate downstream signaling pathways, such as the mitogen‐activated protein kinase (MAPK) cascade, phosphatidylinositol 3‐kinase (PI3K)‐Akt kinase (AKT) cascade, and Janus kinase (JAK)‐signal transducer and activator of transcription (STAT) cascade.

Various negative‐feedback mechanisms have important roles in the regulation of growth factor signaling.^29^, ^30^, ^31^, ^32^ For example, the direct activation of SH2 domain‐containing protein phosphatases, Shp1 and Shp2, serves as negative regulators through dephosphorylation of positive signaling proteins activated by epidermal growth factor receptor (EGFR). Protein kinase C (PKC) is also activated by EGFR through phospholipase Cγ (PLCγ). The activated PKC phosphorylates T654 of EGFR, eliminating the high‐affinity binding site of EGF in the EGFR, serving as another negative‐feedback mechanism. Ubiquitination is another layer of RTK regulation driving their endocytosis, recycling, and degradation via lysosomes.^33^, ^34^ The best‐studied ubiquitin ligating enzyme (E3 ubiquitin ligase) family involved in the negative regulation of RTKs is Casitas B‐lineage lymphoma (Cbl) family.^33^ Cbl family members bind to a phosphotyrosine‐containing peptide in activated RTKs through the N‐terminal region referred to as the tyrosine kinase‐binding domain. Interference with the ubiquitination of RTKs by Cbl family causes impaired intracellular degradation of RTKs, including EGFR, platelet‐derived growth factor receptor (PDGFR), fibroblast growth factor receptor (FGFR), and vascular endothelial growth factor receptor.^30^, ^33^ The Cbl family protein Cbl‐b also inhibits insulin growth factor (IGF) signaling through degradation of insulin receptor substrate 1, the important hub protein activated by IGF receptor (IGFR).^35^ Thus, the Cbl family is involved in the negative regulation of various growth factor signals.

## 2 Basics of Primary Cilia

Primary cilia have three compartments, the basal body, the transition zone, and the axoneme.^1^, ^2^, ^3^, ^4^, ^5^, ^6^, ^7^ The basal body is derived from the mother centriole and has distal and subdistal appendages and docks to the apical plasma membrane through the distal appendage. The axoneme consists of nine circularly arranged microtubule doublets and forms a projection extending from the basal body. The transition zone is a short (0.5 µm) area located above the basal body, characterized by Y‐shaped connectors between the microtubule doublets and primary cilia membrane. Although primary cilia are nonmotile, the formation is dynamically regulated. In 1979, Tucker et al. found that primary cilia were assembled when cultured mouse 3T3 fibroblasts exited the cell cycle under serum deprivation (i.e., G0 phase).^36^, ^37^ They also reported that when the quiescent fibroblasts were stimulated with serum, primary cilia were disassembled after the serum stimulation,^36^, ^37^ which is also the case for RPE1 cells (an immortalized cell line derived from human retinal pigment epithelium).^38^ Deciliation after serum stimulation corresponded to the G0/G1 transition.^38^ Subsequent analyses revealed that the progression to S phase after cell cycle reentry was delayed and shortened if primary cilia were longer and shorter in the G0 phase, respectively.^39^, ^40^ In contrast, forced ciliation in growing cells resulted in the arrest of cell‐cycle progression.^25^, ^26^, ^41^, ^42^, ^43^ These findings suggest that primary cilia themselves can work as negative regulators of the cell cycle. In the following sections, we describe the primary cilia dynamics with an eye on (i) the assembly of primary cilia responding to serum withdrawal, (ii) the disassembly of primary cilia responding to serum stimulation, and (iii) suppression of ciliogenesis in the presence of serum (growth factors).

## 3 Assembly of Primary Cilia Responding to Serum Withdrawal

Two pathways to generate primary cilia, namely, the extracellular and intracellular pathways, have been demonstrated in the assembly of primary cilia responding to serum withdrawal.^6^, ^44^, ^45^, ^46^ In the extracellular pathway, the mother centriole first docks to the plasma membrane, after which axonemal microtubules are nucleated and the cilia grow directly in the extracellular setting. Centrosomal protein 83 (CEP83), which is localized at the distal appendage of the mother centriole, plays important roles in centriole‐to‐membrane docking^47^ and the recruitment of intraflagellar transport 20 (IFT20) protein, which is mandatory for axoneme formation,^48^ to the basal body.^49^ The extracellular pathway‐dependent ciliogenesis is frequently observed in epithelial cells.^45^, ^46^

The current understanding of the intracellular pathway of ciliogenesis can be summarized as follows. First, small cytoplasmic vesicles are transported from the Golgi apparatus to the mother centriole, along using microtubule and actin networks in kinesin/dynein and myosin‐dependent manners, respectively, resulting in the formation of a ciliary vesicle and the conversion from the mother centriole to the basal body.^50^, ^51^ Centrosomal protein 164 (CEP164), which is localized at the distal appendage of the mother centriole, is indispensable for the docking of vesicles at the mother centriole.^52^, ^53^ Second, the basal body is driven toward the plasma membrane by mechanical forces produced during the remodeling of the cytoskeleton induced by serum deprivation and anchored to the plasma membrane via the distal appendage.^54^ Third, tau tubulin kinase (TTBK2) is recruited to the distal appendage, which depends on the decrease in phosphatidylinositol 4‐phosphate (PtdIns4P) after serum withdrawal, and triggers the removal of coiled‐coil protein 110 (CP110) in the inhibitory complex of ciliogenesis, resulting in the initiation of axoneme elongation.^55^, ^56^, ^57^ CP110 associates with centrosomal protein (CEP97), centrosomal protein 290 (CEP290), and Talpid3 and acts as a cap at the distal end of centrioles to block the conversion from the mother centriole to the basal body.^55^, ^58^, ^59^ When cells exit the cell cycle by serum withdrawal, TTBK2 is recruited to the distal end of centrioles, where it removes CP110 from the inhibitory complex of ciliogenesis, possibly through phosphorylating one or more of the proteins in the CP110/CEP97/CEP290/Talpid3 complex.^56^, ^60^ The removal of CP110 is also regulated by neuralized E3 ubiquitin protein ligase 4 (NEURL4).^61^, ^62^ NEURL4 localizes to the daughter centriole in cycling RPE1 cells. Upon serum deprivation, NEURL4 translocates from daughter to mother centriole with the help of cohesion factors, such as leucine‐rich repeat‐containing protein 45 and centrosomal protein 68, at the proximal end of the mother centriole. The translocated NEURL4 facilitates the removal of CP110, possibly through the interaction with HECT‐type E3 ligase HERC2 and triggers ciliogenesis.^62^, ^63^ Fourth, the ciliary vesicle fuses with the plasma membrane. Vast amounts of tubulin are then transported from the cytoplasm into primary cilia by IFT and undergo various post‐translational modifications, including acetylation, detyrosination, and glutamylation, which result in increased axoneme length.^64^, ^65^ The intracellular pathway‐dependent ciliogenesis is frequently observed in fibroblasts, smooth muscle cells, and neurons.^45^, ^46^

The assembly of primary cilia responding to serum withdrawal is also induced by inhibition of Aurora A (AurA), one of the most important mitotic kinases for cell cycle control, which is also involved in the disassembly of primary cilia (described in the following sections). AurA associates with nudE neurodevelopment protein 1 (NDE1), oral‐facial‐digital syndrome 1 (OFD1), and centrosomal P4.1‐associated protein (CPAP) as a ciliary disassembly complex where CPAP works as a scaffold protein.^43^ During G1, NDE1 localizes at the basal body and suppresses ciliogenesis by tethering dynein light chain 1 (DYNLL1), which is usually associated with retrograde IFT components,^66^ at the basal body in 3T3 and RPE1 cells.^39^ When these cells exit the cell cycle by serum deprivation, cyclin‐dependent kinase 5 is activated and phosphorylates NDE1. The phosphorylated NDE1 is recognized and ubiquitylated by F‐box and WD repeat domain‐containing 7 (FBXW7), which is associated with an adaptor protein, S‐phase kinase associated protein 1 (SKP1), a scaffold protein, Cullin 1 (Cul1), and RING‐box protein 1 (Rbx1) as an E3 ligase complex called SCF^FBXW7^. The ubiquitinated NDE1 is then degraded in proteasome, which inhibits the formation of the ciliary disassembly complex, resulting in ciliogenesis.^67^, ^68^

## 4 Disassembly of Primary Cilia Responding to Serum Stimulation

When quiescent mouse 3T3 fibroblasts or human RPE1 cells are stimulated with serum, primary cilia disassemble after serum stimulation.^36^, ^37^, ^38^ Deciliation after serum stimulation corresponds to the G0/G1 transition.^38^ AurA has important roles in the deciliation.^69^ AurA is activated by serum stimulation through Ca^2+^/calmodulin signaling, the noncanonical wingless (WNT) pathway, and phosphatidyl inositol signaling.^12^, ^70^, ^71^, ^72^ Calmodulin activated by Ca^2+^ binds to multiple motifs on AurA and activates it.^73^ Neural precursor cells express developmentally downregulated protein 9 (NEDD9), which is activated via the noncanonical WNT pathway and binds to and activates AurA.^74^ Inositol polyphosphate‐5‐phosphatase E stimulates autophosphorylation and activates AurA, probably through producing phosphatidylinositol‐(3,4)‐diphosphate (PtdIns(3,4)P_2_).^72^ The activated AurA phosphorylates itself and targets proteins during G1, which stimulate the disassembly of primary cilia.^12^ One important substrate of AurA is histone deacetylase 6 (HDAC6). Phosphorylated HDAC6 deacetylates α‐tubulin and reduces the stability of axoneme microtubules.^38^ Several studies support the role of HDAC6 in the disassembly of primary cilia.^75^, ^76^ However, mice lacking HDAC6 develop normally,^77^ suggesting that there may be other important pathways regulated by AurA in the disassembly of primary cilia.

## 5 Suppression of Ciliogenesis in Growing Cells

**Figure 2:**
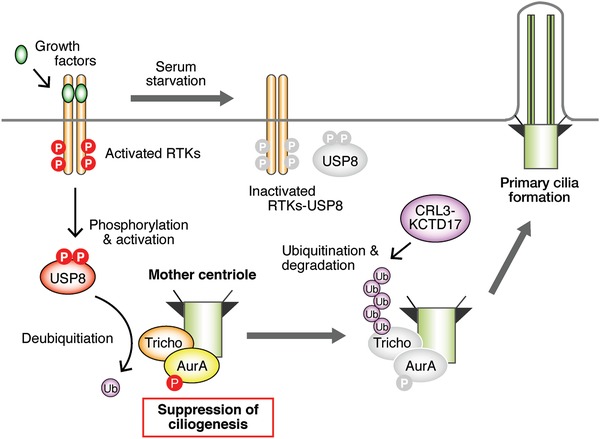
Suppression of ciliogenesis in the presence of growth factors. Activation of RTKs by growth factors phosphorylates USP8, which suppresses the degradation of trichoplein and causes phosphorylation and activation of AurA, resulting in the suppression of ciliogenesis. Serum starvation inactivates RTKs and USP8, which causes degradation of trichoplein that is ubiquitinated by CRL3^KCTD17^ and destabilization of AurA, resulting in ciliogenesis.

We have recently found that knockdown of trichoplein, a centriolar protein originally identified as a keratin‐binding protein (and now as an activator protein of AurA),^78^, ^79^ caused ciliogenesis through the inactivation of AurA and cell cycle arrest in RPE1 cells in the presence of serum.^41^ This experimental setting was quite different from that of ciliogenesis induced by serum starvation. These effects were prevented by simultaneous knockdown of IFT20 or CEP164, suggesting that the ciliogenesis is required for the cell cycle arrest induced by knockdown of trichoplein.^41^ This was the first report indicating that ciliogenesis could inhibit the cell cycle and that the trichoplein‐AurA pathway could be an important player in ciliogenesis‐induced cell cycle arrest. Recently, we analyzed the molecular mechanisms of the regulation of the trichoplein‐AurA pathway (**Figure** **2**).

Given that trichoplein was stabilized by proteasome inhibitors in serum‐starved RPE cells, we performed global screenings of E3 ligases and revealed that potassium channel tetramerization domain‐containing 17 (KCTD17), associated with the scaffold protein Cullin 3 (Cul3) and RBX1 as an E3 ligase complex called CRL3^KCTD17^, was the E3 ligase that could polyubiquitinate trichoplein.^25^ Knockdown of KCTD17 stabilized trichoplein, sustained the activity of AurA, and suppressed the ciliogenesis in RPE1 cells after serum deprivation.^25^ Since the activity of CRL3^KCTD17^ was constant regardless of the presence or absence of serum, we hypothesized the existence of deubiquitinases (DUBs) that could deubiquitinate trichoplein dependent on the serum. Using global screenings of DUBs and their subsequent characterization, we revealed that ubiquitin‐specific peptidase 8 (USP8) could deubiquitinate and stabilize trichoplein and that the DUB activity of USP8 was regulated by EGFR tyrosine kinase through phosphorylation of tyrosine residues at 717 and 810 of USP8.^26^ Other RTKs, including EGFR, PDGFRα, or PDGFRβ, and FGFR1, were also capable of inducing the phosphorylation‐mediated USP8 activation in vitro.^26^ Knockdown of EGFR or USP8 interfered with the trichoplein‐AurA pathway, induced unscheduled ciliogenesis, and caused cell cycle arrest of RPE1 cells, even in the presence of serum. These effects were alleviated when ciliogenesis was abrogated by depletion of IFT20 or CEP164.^26^ These findings suggest that EGF signaling regulates not only the well‐known kinase cascades, such as MAPK and PI3K‐AKT cascades, but also the dynamics of primary cilia.

The balance between ubiquitination and deubiquitination of trichoplein is also modulated by NDE1‐like 1 (NDEL1),^42^ a modulator of dynein activity localized at the subdistal appendage of the mother centriole.^80^, ^81^, ^82^ NDEL1 indirectly inhibits the ubiquitination of trichoplein by CRL3^KCTD17^ and suppresses ciliogenesis in the presence of serum, whereas NDEL1 is degraded via the ubiquitin–proteasome system (UPS) in the absence of serum, resulting in the disappearance of trichoplein at the mother centriole and ciliogenesis.^42^ The molecules involved in the UPS‐mediated NDEL1 degradation remain to be identified.

## 6 PDGF Signaling Regulates Migration through Primary Cilia

Primary cilia and RTKs, especially PDGFRα, play critical roles in cell migration.^15^, ^18^, ^22^ When tissue is wounded, PDGF‐AA, a dimeric glycoprotein composed of two A subunits of PDGF, is secreted mainly from platelets. The PDGF‐AA binds to PDGFRα located in primary cilia in dermal fibroblasts, which causes the autophosphorylation of PDGFRα, resulting in the activation of MEK1/2‐ERK1/2‐p90 ribosomal S6 kinase (p90^RSK^)‐Na^+^/H^+^ exchanger (NHE1) and PI3K‐AKT‐ NHE1 pathways. If primary cilia are defective, these signaling modules are not activated, suggesting that localization of PDGFRα in primary cilia is critical in response to PDGF‐AA to regulate cell migration. The activated NHE1 translocates to the leading and mobile edge of the cell (called the lamellipodium) and organizes the actin cytoskeleton with Ezrin/Radixin/Moesin proteins, which enable directional cell migration. The localization of PDGFRα is regulated by Cbl E3 ubiquitin ligases.^24^ Both c‐Cbl and Cbl‐b interact with IFT20 and ubiquitinate PDGFRα in response to PDGF‐AA stimulation, resulting in the internalization of PDGFRα for negative regulation. If primary cilia are impaired by the depletion of IFT20, both c‐Cbl and Cbl‐b are ubiquitinated and degraded, resulting in the overactivation of PDGFRα localized in the plasma membrane in response to the ligand activation.^24^ PDGF signaling in primary cilia has been excellently reviewed.^15^, ^21^, ^22^

## 7 EGF Signaling Regulates Mechanosensation through Primary Cilia

**Figure 3:**
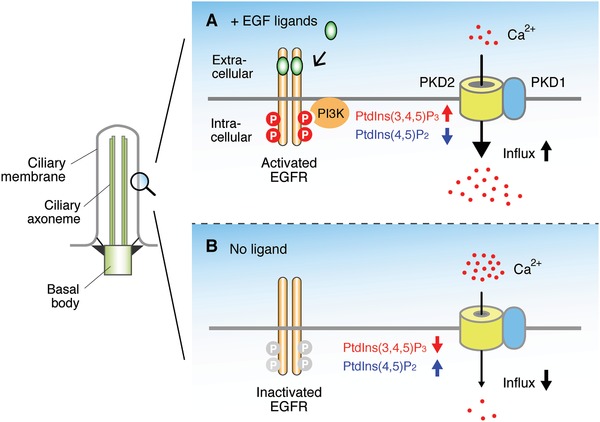
EGF signaling connects mechanosensation to primary cilia. A) When EGF signaling is activated, PtdIns(4,5)P_2_ is phosphorylated by PI3K, which decreases and increases the local concentration of PtdIns(4,5)P_2_ and PtdIns(3,4,5)P_3_, respectively, resulting in the activation of PKD2. B) When EGF signaling is absent, the local concentration of PtdIns(4,5)P_2_ and PtdIns(3,4,5)P_3_ is increased and decreased, respectively, resulting in the suppression of PKD2. By changing the sensitivity of PKD2, EGF signaling regulates mechanosensation.

EGF signaling regulates not only cell proliferation but also the development of various tissues and the maintenance of body homeostasis. In the kidney, EGFR is localized in the primary cilia of epithelial cells of the thick ascending loop of Henle and the distal convoluted tube within the vicinity of polycystin 2 (PKD2).^83^, ^84^, ^85^ PKD2 belongs to the transient receptor potential superfamily of channel proteins and conveys extracellular stimuli to ion, mainly Ca^2+^, currents.^14^, ^86^, ^87^ EGFR in kidney epithelial cells is activated by various EGF ligands during tubulogenesis.^88^, ^89^, ^90^, ^91^, ^92^ The activation of EGFR causes a decreased local concentration of phosphatidylinositol (4,5)‐biphosphate (PtdIns(4,5)P_2_) partly through the phosphorylation of PIP_2_ into phosphatidylinositol (3,4,5)‐triphosphate (PtdIns(3,4,5)P_3_) by PI3K and the hydrolysis of PIP_2_ by PLCγ^85^, ^93^ (**Figure** **3**). The function of PKD2 is suppressed by PtdIns(4,5)P_2_. When EGF signaling is activated, the local concentration of PtdIns(4,5)P_2_ is decreased, which causes activation of PKD2, resulting in increased Ca^2+^ influx.^85^, ^93^ Therefore, EGF signaling can sensitize the primary cilium‐based mechanosensation by reducing the threshold of PKD2 for activation by mechanical stimulation.^87^ Targeted disruption of genes involved in EGF signaling, including EGFR,^94^, ^95^ PKD2,^96^ and USP8,^26^ causes cystic kidney in animal models. The localization of EGFR and PKD2 in primary cilia is also observed in human airway smooth muscle cells^97^ and mouse odontoblasts.^98^ The interaction between EGFR and PKD2 may be involved in the mechanosensation that is necessary for directed migration of these cells.

## 8 TGFβ Signaling Is Involved in Left‐Right Asymmetry Regulated by Primary Cilia

**Figure 4:**
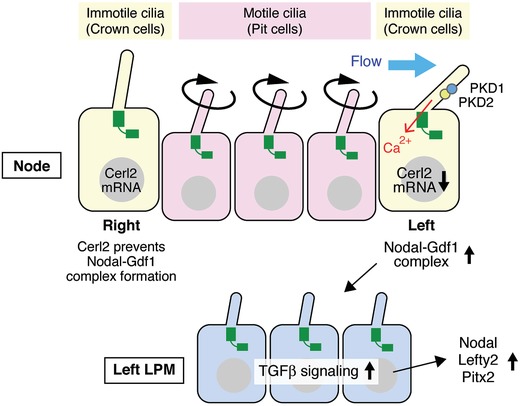
TGFβ signaling is involved in left‐right asymmetry regulated by primary cilia. The ventral node of the mammalian embryo or an equivalent structure in other vertebrates consists of pit cells and perinodal (crown) cells. Cerl2, Nodal, and Gdf1 proteins are expressed bilaterally in the crown cells. When the immotile cilia of crown cells sense the leftward flow generated by the rotational movement of motile cilia of pit cells, the Cerl2 mRNA is degraded on the left side crown cells, which causes the formation of Nodal‐Gdf1 heterodimers. The Nodal‐Gdf1 heterodimers travel to the left side of the LPM and bind to TGFβ receptors, resulting in left‐side morphogenesis through the expression of target genes, including Lefty2 and Pitx2.

Although the human body is externally symmetrical, the visceral organs are arranged asymmetrically in a stereotyped manner.^99^ For example, the heart, spleen, and pancreas are located on the left side of the body, whereas the liver and gall bladder are located on the right side. The left‐right (L‐R) asymmetry is generated through the three steps: (i) the initial breakage of the L‐R symmetry at the ventral node of the mammalian embryo or an equivalent structure of other vertebrates, such as Kupffer's vesicle in zebrafish; (ii) transfer of the L‐R biased signal from the node to the lateral plate mesoderm (LPM), which also has primary cilia,^100^ resulting in the expression of transforming growth factor β (TGFβ)‐related proteins, such as Nodal and Lefty2 on the left side of the LPM; and (iii) L‐R asymmetric morphogenesis of the visceral organs induced by these signaling proteins.^101^ Nodal protein is expressed bilaterally in perinodal cells located at the periphery of the node (crown cells).^102^ When the nonmotile cilia of crown cells sense the leftward flow generated by the rotational movement of motile cilia of pit cells, which are located in the node as an epithelial sheet of a few hundred monociliated cells, the mRNA for Cerberus‐like 2 (Cerl2), an antagonist of Nodal, is degraded crown cells^103^, ^104^, ^105^ located at the left side (**Figure** **4**). Cerl2 usually binds to Nodal and prevents the formation of a heterodimer composed of Nodal and growth differentiation factor‐1 (Gdf1).^103^ Gdf1, another TGFβ‐related factor, is expressed in crown cells and is essential for Nodal expression in LPM.^106^ If the Cerl2 protein is reduced in the left side crown cell, Nodal forms a heterodimer with Gdf1 and travels to left side of the LPM, where the heterodimer binds to type I and type II TGFβ receptors.^107^ Next, the transcription factor forkhead box protein 1 (FoxH1), interacting with SMAD2/3, is activated and binds to the Nodal‐responsive enhancer region in promoters of FoxH1 target genes, including Nodal itself, Lefty2, and Pitx2.^108^ The promoter of Pitx2 also has a conserved binding sequence for Nhx2, enabling asymmetric expression of Pitx2 longer than those of Nodal and Lefty2.^109^ LPM‐derived cells expressing Pitx2 develop left‐side morphogenesis.^110^ These findings suggest that physiological L‐R asymmetry is disturbed if the function of cilia in pit cells and/or crown cells are impaired. Consistent with this, situs inversus, a type of laterality disorder in which all internal organs are reversed, is observed in ciliopathy, a genetic disorder linked to ciliary dysfunction.^111^, ^112^ It remains unknown whether the primary cilia of crown cells and LPM possess receptors for growth factors, such as EGF and TGFβ.

## 9 IGF Signaling Regulates Corticogenesis through Primary Cilia

**Figure 5:**
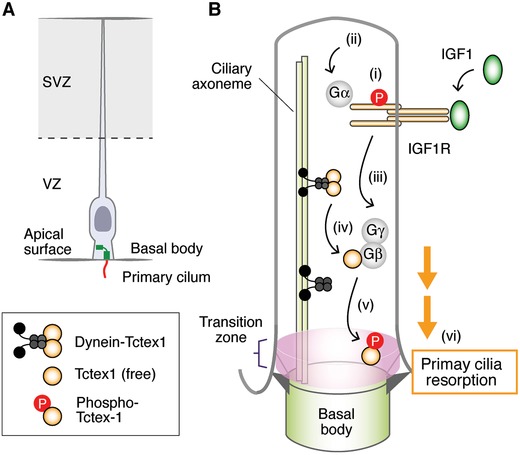
IGF signaling modulates corticogenesis through primary cilia. A) Radial glial cells located in the ventricular zone protrude primary cilia into lateral ventricles filled with cerebrospinal fluid. B) (i) IGF1 secreted into the cerebrospinal fluid from the choroid plexus binds to IGF1R located on primary cilia of radial glial cells and phosphorylates it. (ii) The phosphorylated IGF1R binds to Gα, which (iii) liberates G*βγ*, (iv) triggers the release of Tctex‐1 from the dynein complex, (v) promotes the phosphorylation of Tctex‐1 and recruits the phosphorylated Tctex‐1 to the transition zone. (vi) The phosphorylated Tctex‐1 stimulates the resorption of primary cilia and S phase progression. Shortening G1 increases the proliferation of RG, whereas lengthening G1 stimulates differentiation of radial glial cells into neurons.

The formation of the cerebral cortex begins with the transition from neuroepithelial to radial glial cells, the proliferating progenitors of the developing neocortex, in the ventricular zone^113^ (**Figure** **5**A). The precise coordination between lateral expansion of radial glial cells and differentiation from radial glial to intermediate progenitor cells is critical to form the cerebral cortex. The apical domains of radial glial cells face lateral ventricles filled with cerebrospinal fluid into which the primary cilia of radial glial cells protrude. IGF2 is secreted into the cerebrospinal fluid from the choroid plexus, a highly vascularized tissue located in each ventricle of the brain, and binds to IGF1R located on the primary cilia of radial glial cells.^114^ The binding of IGF2 to IGF1R causes the proliferation of radial glial cells.^115^ The level of IGF2 is highest in late neocortical development, suggesting that IGF2 may preferentially regulate upper‐layer corticogenesis.^115^ IGF1 in cerebrospinal fluid also contributes to the proliferation of radial glial cells^116^ (Figure 5B). The binding of IGF1 to IGF1R on the primary cilia of radial glial cells activates and phosphorylates IGF1R. Although IGF1R is known as a RTK, it also has noncanonical G protein‐coupled receptor activity.^117^, ^118^ The activated IGF1R binds to Gα, releasing G*βγ*. The free G*βγ* triggers the release of Tctex‐1 from the dynein complex, enabling subsequent phosphorylation and recruiting of the phosphorylated Tctex‐1 to the transition zone of the primary cilia of radial glial cells at the ventricular zone.^116^ The phosphorylated Tctex‐1 stimulates the resorption of primary cilia and subsequent S phase progression.^119^ Shortening G1 increases the proliferation of radial glial cells, whereas lengthening G1 stimulates differentiation of radial glial cells into neurons.^120^, ^121^ These findings suggest that impairment of these IGF signaling events may cause abnormal corticogenesis and neurodevelopmental diseases. In fact, microcephaly and mental retardation have been observed in the patients suffering from mutations in IGF1 or IGF1R.^122^, ^123^ The mutation of ADP‐ribosylation factor‐like protein 13B, a causative gene of Joubert syndrome, which is accompanied by autism,^124^, ^125^ disrupts the localization of IGF1R in primary cilia of radial glial cells and impairs the migration and placement of interneurons in the developing cerebral cortex.^126^, ^127^

## 10 Leptin Signaling Regulates Appetite through Primary Cilia

**Figure 6:**
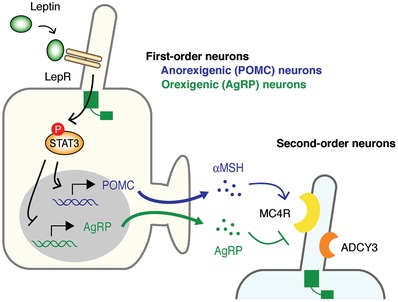
Leptin signaling modulates appetite through primary cilia. The arcuate nucleus is composed of different types of ciliated neurons, including anorexigenic neurons expressing POMC and orexigenic neurons expressing AgRP. If leptin binds to its receptor in ciliated neurons, the transcription of POMC and AgRP is increased and decreased, respectively, through the JAK‐STAT3 pathway. αMSH, cleaved from POMC, also acts as the anorexigenic neuropeptide. MC4R, a common receptor for αMSH and AgRP, and ADCY3 are localized in ciliated PVN neurons expressing SIM1. αMSH and AgRP activate and inhibit MC4R, respectively. ADCY3 is bound to Gs in primary cilia. Inhibition of Gs in SIM1‐expressing neurons is sufficient to cause obesity.

Ciliopathies, such as Bardet‐Biedl syndrome (BBS) and Alström syndrome (ALMS), often accompany obesity, suggesting that the impairment of primary cilia may be involved in the pathogenesis of obesity.^10^, ^11^, ^128^ Obesity results from excessive calorie intake relative to the energy expenditure. The arcuate nucleus of the hypothalamus is the center that regulates calorie intake and energy expenditure. The arcuate nucleus is composed of different types of ciliated neurons, including anorexigenic neurons expressing pro‐opiomelanocortin (POMC) and orexigenic neurons expressing Agouti‐related peptide (AgRP) (**Figure** **6**). POMC is cleaved by proteases to generate α‐melanocyte‐stimulating hormone (αMSH), which is the anorexigenic neuropeptide. These neurons express the leptin receptor in primary cilia.^129^ If leptin, a hormone secreted from adipocytes in response to food intake, binds to its receptor, the transcription of POMC and AgRP is increased and decreased, respectively, through the JAK‐STAT3 pathway.^130^, ^131^ The negative‐feedback system of appetite does not work if primary cilia are ablated by knockout of IFT88 or KIF3A, suggesting the fundamental role of primary cilia in appetite regulation by leptin.^10^, ^132^ Genes associated with BBS and ALMS encode ciliary proteins, and the mutated proteins impair the function of primary cilia in hypothalamic neurons, which is the potential cause of obesity in these disorders.^133^

POMC/αMSH and AgRP‐producing neurons in the arcuate nucleus send their axonal projections to second‐order neurons in the paraventricular nucleus (PVN). Melanocortin 4 receptor (MC4R), a common receptor for αMSH and AgRP, is strongly expressed in the ciliated PVN neurons expressing single‐minded 1 (SIM1), where αMSH and AgRP activate and inhibit MC4R, respectively.^134^, ^135^, ^136^, ^137^ Mutations of MC4R account for 3–5% of all severe cases in human obesity.^138^, ^139^, ^140^ MC4R is a G protein‐coupled receptor (GPCR) coupled with Gα stimulatory subunit (Gs).^141^ Mutations of adenylate cyclase 3 (ADCY3) have also been associated with obesity.^142^, ^143^, ^144^, ^145^ ADCY3 is bound to Gs in primary cilia.^146^ MC4R and ADCY3 are localized in the primary cilia of SIM1‐expressing neurons.^137^ Obesity‐associated mutations in MC4R cause impairment of the localization in the primary cilia of SIM1‐expressing neurons. Inhibition of Gs in SIM1‐expressing neurons is sufficient to cause obesity. These findings suggest that the impairment of primary cilia of SIM1‐expressing neurons may be a common mechanism underlying at least some genetic causes of human obesity.^137^

## 11 Hedgehog Signaling Regulates Cancer Cell Proliferation through Primary Cilia

**Figure 7:**
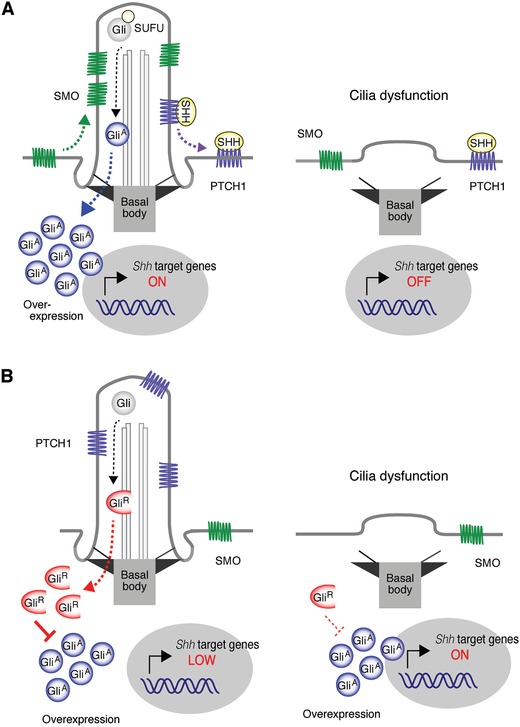
Hedgehog signaling relates cancer cell proliferation through primary cilia. In cancer cells with amplification of SHH, SHH binds to PTCH1, which causes the accumulation of SMO in primary cilia, resulting in the conversion of Gli to Gli^A^ and translocation of Gli^A^ to nucleus. The transcription of SHH target genes is activated by Gli^A^. A) If the primary cilium is lost, the cilium‐dependent conversion of Gli^A^ is inhibited, resulting in the suppression of proliferation. In cancer cells with amplification of Gli, the amount of Gli^A^ can increase without the help of SMO (independent of primary cilia). B) In the absence of SHH, primary cilia increase the conversion of Gli to Gli^R^, which inhibits the transcription of SHH target genes. Primary cilia can A) promote and B) suppress the tumorigenesis, depending on the context.

Although primary cilia are lost in a wide range of cancer types, as described in the section below, primary cilia can also promote tumor progression in different types of cancer, including medulloblastoma, basal cell skin cancer, and basal‐like breast cancer.^2^, ^147^, ^148^, ^149^ Medulloblastoma comprises four major subgroups: sonic hedgehog (SHH), WNT, group 3, and group 4.^150^, ^151^, ^152^ The SHH subgroup accounts ≈30% of all cases. In the SHH groups, somatic mutations and amplifications of genes involved in hedgehog pathway have been identified, including Patched (PTCH1), SMO, suppressor of fused (SUFU), and Gli transcription factor 2 (GLI2).^153^ SHH is a secretory protein that binds to the receptor PTCH1. In the absence of SHH, PTCH1 is located in primary cilia and keeps SMO, a seven‐pass transmembrane protein, outside primary cilia. When SHH binds to PTCH1, it disappears from primary cilia, allowing accumulation of SMO in primary cilia (**Figure** **7**A). SUFU is also accumulated in primary cilia in the presence of SHH. There are three Gli transcription factors: GLI1, GLI2, and GLI3. GLI2 and GLI3 can be converted to transcriptional activators or repressors, depending on their proteolytic processing. GLI1 is a transcriptional target of GLI2 and GLI3 and acts as a transcriptional activator. In the absence of SHH, GLI2 and GLI3 (predominant) are converted to transcriptional repressors (GLI2‐R and GLI3‐R) in primary cilia and translocate to the nucleus, resulting in the suppression of hedgehog signaling (Figure 7B). In the presence of SHH, both GLI2 and GLI3 are converted to transcriptional activators (GLI2‐A and GLI3‐A) in primary cilia and translocate to the nucleus, where they mediate hedgehog signaling at the level of transcription (Figure 7A). SMO and SUFU accumulated in primary cilia are positively and negatively involved in the conversion and translocation of GLI2/3‐A, respectively.^147^, ^148^, ^154^, ^155^ Loss‐of‐function mutation in PTCH1 or SUFU and gain‐of‐function mutation in SMO or GLI2 can enhance hedgehog signaling, resulting in tumorigenesis. Because PTCH1 and SMO work in primary cilia to generate GLI2‐A and GLI3‐A, primary cilia promote the progression of SHH medulloblastoma caused by mutations of PTCH1 and SMO. In the case of mutation or amplification of GLI2, the amount of GLI2‐A can increase without the help of PTCH1 and SMO (e.g., cilia‐independent) (Figure 7B). In this case, GLI3‐R is predominantly generated in primary cilia in the absence of SHH and antagonizes GLI2‐A. Therefore, primary cilia suppress the progression of SHH medulloblastoma caused by the mutation/amplification of GLI2.^156^ The dual and opposing roles of primary cilia are also observed in basal cell carcinoma (BCC), a skin cancer caused by dysregulated hedgehog signaling.^157^, ^158^, ^159^ Primary cilia are expressed in BCC caused by activated form of SMO and ablation of primary cilia inhibits the progression of BCC. In contrast, ablation of primary cilia expressed in BCC caused by activated GLI2 accelerates the progression of BCC.^157^ These findings suggest that primary cilia can promote and suppress tumorigenesis depending on the context.

The context‐dependent functions of primary cilia are also related to the resistance mechanism of SMO inhibitors.^160^, ^161^ Two SMO inhibitors, vismodegib and sonidegib, have been approved by the U.S. Food and Drug Administration for treatment in advanced or metastatic BCC. Several other SMO inhibitors have also been developed in clinical trials for treatment in medulloblastoma. Despite good initial responses, mutation‐related resistance to the SMO inhibitors often emerges.^162^ Although most mutations are observed in the drug‐binding pocket of SMO, mutations in genes related to ciliogenesis, including OFD1, IFT80, and retinitis pigmentosa GTPase regulator‐interacting protein 1 (RPGRIP1), have also been identified.^160^ The mutations in ciliary genes cause the loss of primary cilia on SHH medulloblastoma cells and confer resistance to SMO inhibitors due to the lack of GLI‐R and the presence of full‐length GLI2, the latter of which can work as a weak transcriptional activator without SMO signaling.^160^ Additional mutations, such as loss‐of‐function mutations in SUFU, increase the nuclear transport of GLI2 and enhance hedgehog signaling, resulting in the aggressive growth of medulloblastoma and BCC resistant to SMO inhibitors.^160^, ^163^, ^164^ Several strategies can be considered to overcome this drug resistance, including the inhibition of GLI‐A and reintroduction of primary cilia.^160^, ^161^ Small molecules have been identified that inhibit GLI‐A^162^, ^165^, ^166^, ^167^, ^168^, ^169^, ^170^, ^171^ and reintroduce primary cilia.^172^, ^173^

Hedgehog signaling in cancer can be regulated by the UPS.^174^, ^175^, ^176^ β‐Transducin repeat‐containing E3 ubiquitin protein ligase (β‐TRCP) is an F‐box protein associated with an adaptor protein, SKP1, a scaffold protein, Cul1, and RING protein Rbx1, which together act as an E3 ligase complex.^177^, ^178^ β‐TRCP recognizes a consensus DSG(X)_2+_ *_n_*S degron in most of its substrates, including GLI2, GLI3, WW domain‐containing transcription regulator 1 (WWTR1), β‐catenin, programmed cell death 4 (PDCD4), and NFκB inhibitor (IκB).^179^, ^180^ In the absence of SHH, the serine residues of the DSG(X)_2+_ *_n_*S degron in GLI2 and GLI3 are phosphorylated by protein kinase A, glycogen synthase kinase 3, and casein kinase 1. β‐TRCP recognizes the phosphorylated degron and processes GLI2/3 to GLI2/3‐R, resulting in the suppression of hedgehog signaling.^174^, ^175^, ^181^, ^182^, ^183^, ^184^ β‐TRCP also recognizes the phosphorylated degrons in TAZ and β‐catenin and degrades these oncogenic proteins, suggesting that β‐TRCP can work as a tumor suppressor.^180^, ^185^ Consistent with this idea, somatic mutations of β‐TRCP are observed in gastric cancer.^186^, ^187^ However, overexpression of β‐TRCP is also reported in various cancers.^177^ In fact, β‐TRCP also recognizes the phosphorylated degrons in PDCD4 and IκB and degrades these oncosuppressor proteins.^188^, ^189^ Therefore, it is conceivable that β‐TRCP can act as a tumor suppressor and oncoprotein, depending on the cellular and extracellular context.^180^

Primary cilia disappear from growing cells, even in the context of tumors where progression is positively correlated with the presence of primary cilia.^190^ Therefore, forced ciliogenesis during cell proliferation may be an effective therapeutic approach to stop cancer cell proliferation despite the dependence of primary cilia on progression.

## 12 RTKs, Cancer Cells, and Primary Cilia

Primary cilia are lost in a wide range of cancer types, including clear‐cell renal‐cell carcinoma,^191^, ^192^, ^193^ epithelial ovarian cancer,^16^ cholangiocarcinoma,^194^ pancreatic ductal adenocarcinoma (PDAC),^195^ astrocytoma/glioblastoma,^196^ luminally derived breast cancer,^197^ melanoma,^198^ chondrosarcoma,^199^ and prostate cancer.^200^ These findings and the negative role of primary cilia in cell cycle progression suggest the hypothesis that ciliopathies may predispose tissue to the development of cancer. In fact, renal cancers can be comorbid in two ciliopathies, Birt‐Hogg‐Dube syndrome and VHL syndrome.^201^, ^202^ However, except for these ciliopathies, cancer incidence is not increased in human ciliopathies.^203^ Thus, the relationship between primary cilia and cancer remains to be fully elucidated.

**Figure 8:**
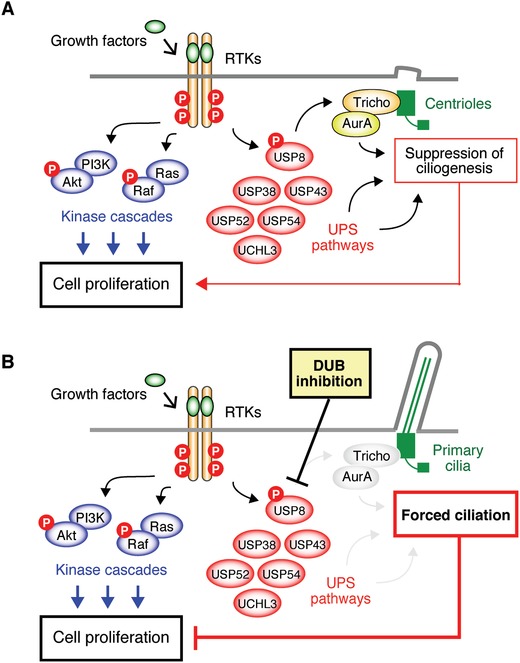
Mechanisms underlying the loss of primary cilia frequently observed in cancer cells with aberrant RTK. Activation of RTKs deciliates primary cilia through activation of the USP8‐trichoplein‐AurA pathway. Other USPs, such as USP54 and UCHL3, may be involved in the deciliation induced by activation of RTKs. A) The suppression of ciliogenesis stimulates cell proliferation, which is independent of the stimulation of cell proliferation through the Ras‐Raf and PI3K‐AKT cascades. B) Inhibition of deubiquitinases, such as USP8, suppresses the trichoplein‐AurA pathway, resulting in forced ciliation, which can suppress cell proliferation independently of the Ras‐Raf and PI3K‐AKT cascades.

RTK signaling is also frequently activated in various cancer cells^30^, ^204^ (**Figure** **8**). For example, EGFR family receptors are amplified and/or mutated in various human tumors, including gliomas, non‐small‐cell lung carcinoma, breast, gastric, and ovarian cancers.^205^, ^206^ Amplification or mutation of EGFR‐family receptors can cause overexpression and/or constitutive activation of EGFR signaling in these tumor tissues.^205^, ^206^, ^207^ Activation of EGFR results in the recruitment of Grb2, a SH2 domain protein complexed with the guanine exchange factor son‐of‐sevenless (Sos), to the tyrosine‐phosphorylated EGFR. This recruitment brings Sos close to the small GTPase protein Ras, leading to the activation of Ras by catalyzing the replacement of GDP to GTP. Following Ras activation, members of the Raf family of serine/threonine kinases are recruited to the cell membrane through binding to Ras. The recruited Raf activates mitogen‐activated protein kinase kinase 1 (MEK1) and MEK2 via phosphorylation of multiple serine/threonine residues. MEK1 and MEK2 are tyrosine and serine/threonine dual‐specificity kinases that subsequently activate extracellular signal‐regulated kinase (ERK). Grb2 is also important to activate PI3K‐AKT cascade. Gab1, a docking protein, is recruited to activated EGFR through Grb2 and is phosphorylated at tyrosine residues, where SH2 domains of PI3K bind. The binding of PI3K to Gab1 induces the formation of PtdIns(3,4,5)P_3_, serving as a docking site for proteins that have phospholipid‐binding domains, including AKT. If AKT binds to PIP_3_, it is activated through autophosphorylation on two stimulatory serine/threonine residues. The activation of the Ras‐Raf‐MEK‐ERK and PI3K‐AKT pathways promotes cell proliferation. The recent findings that activation of RTKs, such as EGFR, deciliates primary cilia through activation of a USP8‐trichoplein‐AurA pathway may provide a novel explanation for the loss of primary cilia frequently observed in cancer cells with aberrant RTK signaling.^25^, ^26^

## 13 Future Directions

Growth factor and morphogen signaling through primary cilia are undoubtedly important for both health and disease. Although many mechanisms underlying these signaling events have been elucidated, many questions still exist. The clinical phenotypes observed in ciliopathies are highly heterogeneous, even in the same syndrome, depending on the organ system in which primary cilia are impaired^14^ and the mutation state of the individual patient.^208^ Growth factor and morphogen signaling are highly context dependent.^209^, ^210^, ^211^ In addition, complex crosstalk between various growth factor and morphogen signaling pathways through primary cilia occurs.^15^, ^19^, ^22^ A primary cilium can express various receptors for growth factors and morphogens. Several molecules have been identified as hubs of signaling pathways activated by different growth factors and morphogens at the primary cilium. For example, the expression of Gli2, one of the most important targets of hedgehog signaling, is also increased by TGFβ signaling through a SMAD2/3 and tuberous sclerosis complex protein 1‐dependent pathway in the primary cilia of mouse embryonic fibroblasts.^212^ USP8 is also activated by EGF, PDGF, and FGF signaling.^26^ The complete mechanism by which each growth factor and morphogen signaling pathway contributes to the activation of these hub molecules and how the integrated activity of the hubs is involved in health and disease remain to be elucidated.

Omics approaches, such as transcriptome analysis and mass spectrometry, may be useful tools to reveal the complexity of growth factor and morphogen signaling through primary cilia. For example, transcriptome analysis uncovered that TGFβ signaling that is activated at the ciliary pocket, the proximal part of the primary cilium residing in the cytoplasm within an invagination of the plasma membrane, is associated with differentiation into cardiomyocytes.^17^ Mass spectrometry successfully revealed novel functions of proteins of primary cilia in regulating TGFβ/bone morphogenic protein signaling^23^ and FGF signaling.^213^ Inducible pluripotent stem cells and animal models, such as mice and zebrafish, combined with genome‐editing technology, such as CRISPR/Cas9, can also be used to examine the complex phenotypes induced by the impairment of target genes in primary cilia.^26^, ^214^

The molecular mechanisms underlying the regulation of cell proliferation by growth factors and morphogens through primary cilia remain to be completely elucidated. The recent finding that activation of EGFR induces deciliation through a USP8‐trichoplein‐AurA pathway suggests that inhibition of this pathway is able to block the proliferation of some cells. DUBs (USP8, USP38, USP43, USP52, USP54, or UCHL3) can suppress ciliogenesis.^26^ The expression of USP54 is increased in colorectal cancer and positively associated with poor prognosis. Downregulation of USP54 in this type of cancer cell may suggest that tumor progression is reduced through primary cilium formation.^215^ Whether inhibition of DUBs affects the proliferation of several cancer cells is an interesting area for further examination (Figure 8B). Finally, the downstream signaling proteins of AurA remain largely elusive. Further studies are necessary to completely elucidate the mechanisms by which primary cilia affect cell cycle progression regulated by growth factors and morphogens.

## Conflict of Interest

The authors declare no conflict of interest.
